# Comparing the effectiveness of long-term use of daily and weekly glucagon-like peptide-1 receptor agonists treatments in patients with nonalcoholic fatty liver disease and type 2 diabetes mellitus: a network meta-analysis

**DOI:** 10.3389/fendo.2023.1170881

**Published:** 2023-06-05

**Authors:** Xia Yuan, Zhe Gao, Caixuan Yang, Kaixin Duan, Luping Ren, Guangyao Song

**Affiliations:** ^1^ Department of Internal Medicine, Graduate School of Hebei Medical University, Shijiazhuang, Hebei, China; ^2^ Department of Endocrinology, Hebei General Hospital, Shijiazhuang, Hebei, China; ^3^ Department of Internal Medicine, Graduate School of Hebei North University, Zhangjiakou, Hebei, China

**Keywords:** glucagon-like peptide-1 receptor agonists, nonalcoholic fatty liver disease, type 2 diabetes, liver fat content, alanine aminotransferase, aspartate aminotransferase

## Abstract

**Objective:**

In the present network meta-analysis (NMA), we aimed to compare the effectiveness of daily and weekly treatment with glucagon-like peptide-1 receptor agonists for patients with nonalcoholic fatty liver disease (NAFLD) and type 2 diabetes mellitus (T2DM).

**Method:**

We used Stata 17.0 for the NMA. Eligible Randomized controlled trials (RCTs) were searched in PubMed, Cochrane, and Embase databases until December 2022. Two researchers independently screened the available studies. The Cochrane Risk of Bias tool was used to assess the risk of bias in the included studies. We used GRADEprofiler (version3.6) to analyze the evidence certainty. Primary outcomes such as liver fat content (LFC), aspartate aminotransferase (AST), and alanine aminotransferase (ALT) levels, as well as secondary outcomes such as γ-glutamyltransferase (γGGT) and body weight, were evaluated. Then, each intervention was ranked by the surface under the cumulative ranking curve (SUCRA). As a supplement, we drew forest plots of subgroup using RevMan (version 5.4).

**Results:**

Fourteen RCTs involving 1666 participants were included in the present study. The NMA results showed that exenatide (bid) was the best treatment for improving LFC compared with other agents, liraglutide, dulaglutide, semaglutide (qw) and placebo), and the SUCRA values were 66.8%. Among five interventions (except exenatide (bid) and semaglutide (qw)) evaluated for AST outcome, and six interventions (except exenatide (bid)) evaluated for ALT outcome, semaglutide (qd) was the most effective drug (SUCRA (AST) = 100%, SUCRA (ALT) = 95.6%). The result of LFC in daily group was MD = -3.66, 95% CI [-5.56, -1.76] and in weekly GLP-1RAs group, it was MD = -3.51, 95% CI [-4, -3.02]. As to AST and ALT, the results in daily group versus weekly group were AST: MD = -7.45, 95% CI [-14.57, -0.32] versus MD= -0.58, 95% CI [-3.18, 2.01] and ALT: MD = -11.12, 95% CI [-24.18, 1.95] versus MD = -5.62, 95% CI [-15.25, 4]. The quality of evidence was assessed as moderate or low.

**Conclusion:**

The daily GLP-1RAs may be more effective in primary outcomes. And the daily semaglutide may be the most effective treatment for NAFLD and T2DM among the six interventions.

## 1 Introduction

Nonalcoholic fatty liver disease (NAFLD) refers to the excessive accumulation of fats in the liver caused by factors other than alcohol and drug consumption (1). NAFLD is the most common chronic liver disease, ranging from simple hepatic steatosis to nonalcoholic steatohepatitis (NASH) (2). NAFLD is often closely related to metabolic disorders such as obesity and type 2 diabetes mellitus (T2DM) (3). Moreover, NAFLD is highly likely to progress to cirrhosis and cancer without active intervention, thus reducing the quality of life of patients and leading to psychological and physical burdens. Weight loss remains the basic of treatment for NAFLD and NASH (4). Although weight loss can improve NAFLD, the effect cannot last for an extended period, thus NAFLD requires long-term and adequate treatment with some drugs (5). However, specific drugs for NAFLD are scarce.

Recently, some studies have shown the role of glucagon-like peptide-1 receptor agonists (GLP-1RAs) in NAFLD treatment. GLP-1RAs can control energy intake and weight gain by prolonging gastric emptying and suppressing appetite (6, 7). Furthermore, GLP-1RAs can improve liver enzyme functions and liver steatosis and significantly reduce liver fat content (8–12). Many GLP-1RA preparations are available for selection, which can be divided into daily preparations and weekly preparations according to the frequency of administration. Weekly agents include semaglutide (qw), dulaglutide and exenatide (qw), whereas daily agents include liraglutide, semaglutide (qd), and exenatide (bid), which are commonly used preparations. The elimination half-life of weekly preparations is of several weeks, and their structural peculiarity results in a slow release, thus maintaining effective blood concentrations for a long time, delaying the onset. In contrast, the elimination half-life of daily preparations is shorter, thus providing active circulating concentrations, and effective blood concentrations can be reached earlier (13). Therefore, the efficacies of these two preparations differ. Although GLP-1RAs can significantly improve liver enzyme functions and liver fat content, a comparative study on the effect of weekly and daily GLP-1RAs on NAFLD with T2DM is unavailable.

Thus, in the present network meta-analysis (NMA), we aimed to compare the efficacy of the long-term use of weekly and daily GLP-1RAs for NAFLD with T2DM, hoping to provide a basis for selecting appropriate clinical drugs.

## 2 Material and methods

### 2.1 Search strategy

A search for all treatments in NAFLD was conducted across the PubMed databases from the date of inception until December 2022 using the following search strategy: (Liraglutide OR Dulaglutide OR Semaglutide OR Albiglutide OR Lixisenatide OR Exenatide OR glucagon-like peptide-1 agonists OR glucagon like peptide OR GLP-1 receptor agonists OR glp-1) AND (Non-Alcoholic Fatty Liver Disease OR Nonalcoholic Fatty Liver Disease OR Nonalcoholic Fatty Liver OR NAFLD OR Nonalcoholic Steatohepatitis OR NASH) AND (liver enzymes OR alanine aminotransferase OR aspartate aminotransferase OR γ-glutamyl transferase OR ALT OR AST OR γGGT OR intrahepatic fat content OR liver fat content OR intrahepatic content of lipid OR hepatic lipid content OR hepatic fat content OR LFC OR IHF OR IHCL OR HFC) in all fields without other limitations.

And search strategies for PubMed, Cochrane and Embase databases were shown in **Table 1**.

**Table 1:** Search strategy for each database.

Databases (number of studies)	Search Strategy
PubMed (224)	(Liraglutide OR Dulaglutide OR Semaglutide OR Albiglutide OR Lixisenatide OR Exenatide OR glucagon-like peptide-1 agonists OR glucagon like peptide OR GLP-1 receptor agonists OR glp-1) AND (Non-Alcoholic Fatty Liver Disease OR Nonalcoholic Fatty Liver Disease OR Nonalcoholic Fatty Liver OR NAFLD OR Nonalcoholic Steatohepatitis OR NASH) AND (liver enzymes OR alanine aminotransferase OR aspartate aminotransferase OR γ-glutamyl transferase OR ALT OR AST OR γGGT OR intrahepatic fat content OR liver fat content OR intrahepatic content of lipid OR hepatic lipid content OR hepatic fat content OR LFC OR IHF OR IHCL OR HFC)
Embase (649)	('liraglutide' OR 'dulaglutide' OR 'semaglutide' OR 'albiglutide' OR 'lixisenatide' OR 'exenatide' OR 'glucagon-like peptide-1 agonist' OR 'glucagon like peptide' OR 'glp-1 receptor agonist' OR 'glp-1') AND ('non-alcoholic fatty liver disease' OR 'nonalcoholic fatty liver disease' OR 'nonalcoholic fatty liver' OR 'nafld' OR 'nonalcoholic steatohepatitis' OR 'nash') AND ('liver enzymes' OR 'alanine aminotransferase' OR 'aspartate aminotransferase' OR 'γ-glutamyl transferase' OR 'alt' OR 'ast' OR 'γggt' OR 'intrahepatic fat content' OR 'liver fat content' OR 'intrahepatic content of lipid' OR 'hepatic lipid content' OR 'hepatic fat content' OR 'lfc' OR 'ihf' OR 'ihcl' OR 'hfc')
Cochrane (182)	(“Liraglutide” OR “Dulaglutide” OR “Semaglutide” OR “Albiglutide” OR “Lixisenatide” OR “Exenatide” OR “glucagon-like peptide-1 agonist*” OR “glucagon like peptide*” OR “GLP-1 receptor agonist*” OR “glp-1”) in All Text AND (“Non-Alcoholic Fatty Liver Disease” OR “Nonalcoholic Fatty Liver Disease” OR “Nonalcoholic Fatty Liver” OR “NAFLD” OR “Nonalcoholic Steatohepatitis” OR “NASH”) in All Text AND (“liver enzymes” OR “alanine aminotransferase” OR “aspartate aminotransferase” OR “γ-glutamyl transferase” OR “ALT” OR “AST” OR “γGGT” OR “intrahepatic fat content” OR “liver fat content” OR “intrahepatic content of lipid” OR “hepatic lipid content” OR “hepatic fat content” OR “LFC” OR “IHF” OR “IHCL” OR “HFC”)

### 2.2 Inclusion and exclusion criteria

The paper inclusion criteria were as follows: (1) Subjects: clinically diagnosed as NAFLD or NASH with T2DM; (2) Drug interventions: patients in the experimental group were treated with GLP-1RAs; (3) Study type: randomized controlled trials (RCTs);

The paper exclusion criteria were as follows: (1)Animal models; (2)Duplicate articles; (3) Subjects were aged <18 years; (4) Study duration <12weeks. (5) The outcomes: liver fat content (LFC), aspartate aminotransferase (AST), alanine aminotransferase (ALT), γ-glutamyl transferase (γGGT) and body weight were not clearly reported. (6)The interventions were not GLP-1RAs versus placebo or blank control; (7)Data outcomes could not be extracted.

### 2.3 Study selection and data extraction

Study selection and data extraction were conducted separately by two individuals. Two reviewers initially selected the relevant studies by reading the title and abstract and then selected the studies for NMA based on the inclusion and exclusion criteria and after reading the full text. Next, any disagreements were resolved by discussion or by a third researcher.

The extracted data included: 1) the baseline information: the last name of the first author, publication year, intervention and control, sample size (female/male), dose (frequency of application), duration, baseline age (mean ± standard deviation [SD]), T2DM, with or without NASH, and the countries of study population, the characteristics of included studies were listed in **Table 2**; 2) the data used for analysis: mean and SD changes from the baseline to the end of each outcome, and sample size (n); 3) the information for quality assessment; 4) the items of evidence certainty assessment.

**Table 2:** The characteristics of the included RCTs.

reference	Author and publication year	Treatment and sample size(female/male)		Dose (frequency of application)	duration(W)	Baseline age (mean±SD)	T2DM	NASH(Y/N)	Study Country
(12)	Kuchay 2020	Dula(9/23)	blank control (10/22)	0.75mg(4W)→1.5mg (once-weekly)	24	46.6 ± 9.1vs48.1 ± 8.9	Y	–	India
(14)	Cusi 2018	Dula (307/183)	Placebo(155/115)	1.5mg(once-weekly)	24	55.2 ± 9.6vs55.0 ± 9.7	Y	Y	the USA
(15)	Harreiter 2021	Exe(16)	Placebo(14)	2mg(once-weekly)	24	59.4±8.5vs60.9±7.4	Y	–	Australia
(16)	Hartman 2020	Dula(30/24)	Placebo(22/29)	1.5mg(once-weekly)	26	58.7±7.8vs56.6±8.9	Y	Y	the USA
(17)	Loomba 2022	Sema(31/16)	Placebo(18/6)	0.24mg→2.4mg (once-weekly)	16	59.9±7.1vs58.7±9.7	75 %Y	–	Europe and the USA
(18)	Armstrong 2016	Lira(8/18)	Placebo(13/13)	1.8mg(once-daily)	48	50±11vs52±12	Y	Y	England
(19)	Bizino 2019	Lira(9/14)	Placebo(11/15)	1.8mg(once-daily)	26	60±6vs59±7	Y	–	Europe
(9)	Guo 2020	Lira(15/16)	Placebo(10/20)	1.8mg(once-daily)	26	53.1 ± 6.3vs52.6 ± 3.9	Y	–	China
(20)	Matikainen 2018	Lira(2/13)	Placebo(4/3)	1.8mg(once-daily)	16	62±2vs63±2	Y	–	Europe
(21)	Nahra 2021	Lira(60/50)	Placebo(55/57)	1.8mg(once-daily)	54	55.5±9.8vs57.3 ±9.5	Y	Y	8 countries (Europe, Canada and the USA et.)
(22)	Newsome 2021	Sema(47/35)	Placebo(44/36)	0.4mg(once-daily)	72	54.3±10.2vs52.4±10.8	61 %Y	Y	Europe and the USA
(23)	Smits 2016	Lira(5/12)	Placebo(4/13)	1.8mg(once-daily)	12	60.8±7.4vs65.8±5.8	Y	–	Europe
(24)	Samson 2011	Exe(11)	blank control (10)	5ug(2W)→10ug (twice-daily)	48	52±3	Y	–	USA
(25)	Shahinul 2020	Lira(16)	Placebo(16)	0.6mg(1W)→1.2mg (once-daily)	24	–	Y	–	Bangladesh

### 2.4 Quality assessment and evidence certainty assessment

The Cochrane Risk of Bias tool (26) was used to assess the risk of bias of the included studies. The following seven items were included: 1) “random sequence generation”: describes how the sequence was generated, such as by using a random table of numbers or a computer for generating a random sequence of numbers; 2) “allocation concealment”: whether the subjects and researchers were aware of group assignments, such as through assignment hiding *via* telephone and Internet; 3) “blinding of the participants and personnel”: whether subjects, researchers, and all participants were blinded; 4) “blinding of outcome assessment”: describe whether an outcome assessor was blinded, but objective outcomes, such as serological outcomes, were unlikely to be affected by the lack of blinding; 5) “incomplete outcome data”: whether there was any missing data, such as loss to follow-up and exclusion of data from analysis; 6) “selective reporting”: whether all outcomes were reported; 7) “other bias”: each study was considered to have a “high”, “low”, or “unclear” risk of bias. The judgment of risk of bias was conducted by two authors separately in Review Manager (Version 5.4).

And then, we used GRADE (Grades of Recommendation, Assessment, Development and Evaluation) model to assess the evidence certainty (27). Since all the included studies were RCTs, we evaluated the following five items: 1) risk of bias: such as allocation concealment, blinding and loss to follow-up, and so on; 2) inconsistency: the results heterogeneity, and whether the authors give a reasonable explanation for its high heterogeneity; 3) indirectness; 4) imprecision: whether the confidence interval (CI) was wide and the sample size was large; 5) publication bias: the number of included studies. This assessment was performed in GRADEprofiler (version 3.6).

### 2.5 Statistical analysis

**Figure 1:**
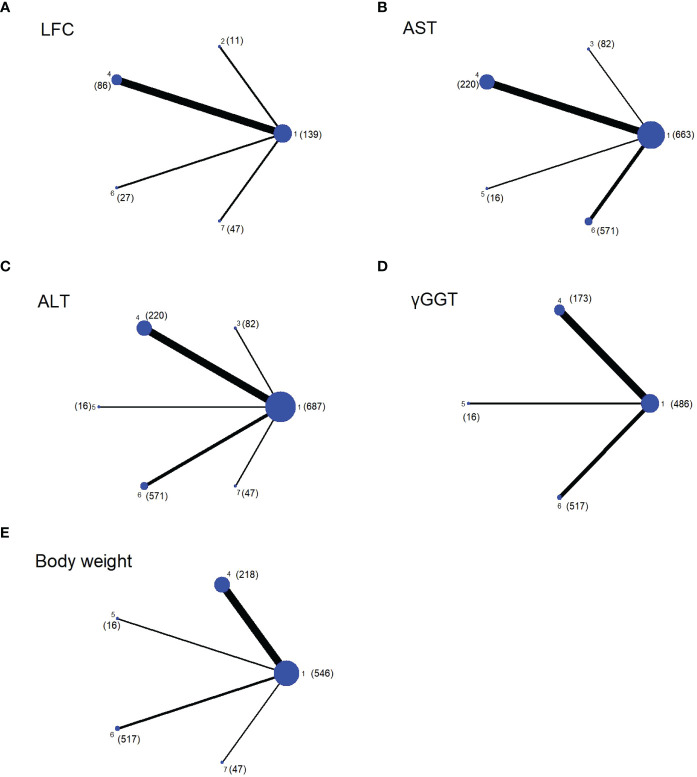
Network plots of evidence for each outcome, with the size of the dots representing the sample size (the specific sample size shown in brackets), and the thickness of the lines representing the number of studies comparing the two interventions. The number mean: 1. Placebo; 2. Exenatide (bid); 3. Semaglutide (qd); 4. Liraglutide; 5. Exenatide (qw); 6. Dulaglutide; 7. Semaglutide (qw). **(A)** Network plot of LFC; **(B)** Network plot of AST; **(C)** Network plot of ALT; **(D)** Network plot of γGGT; **(E)** Network plot of Body weight.

**Figure 2:**
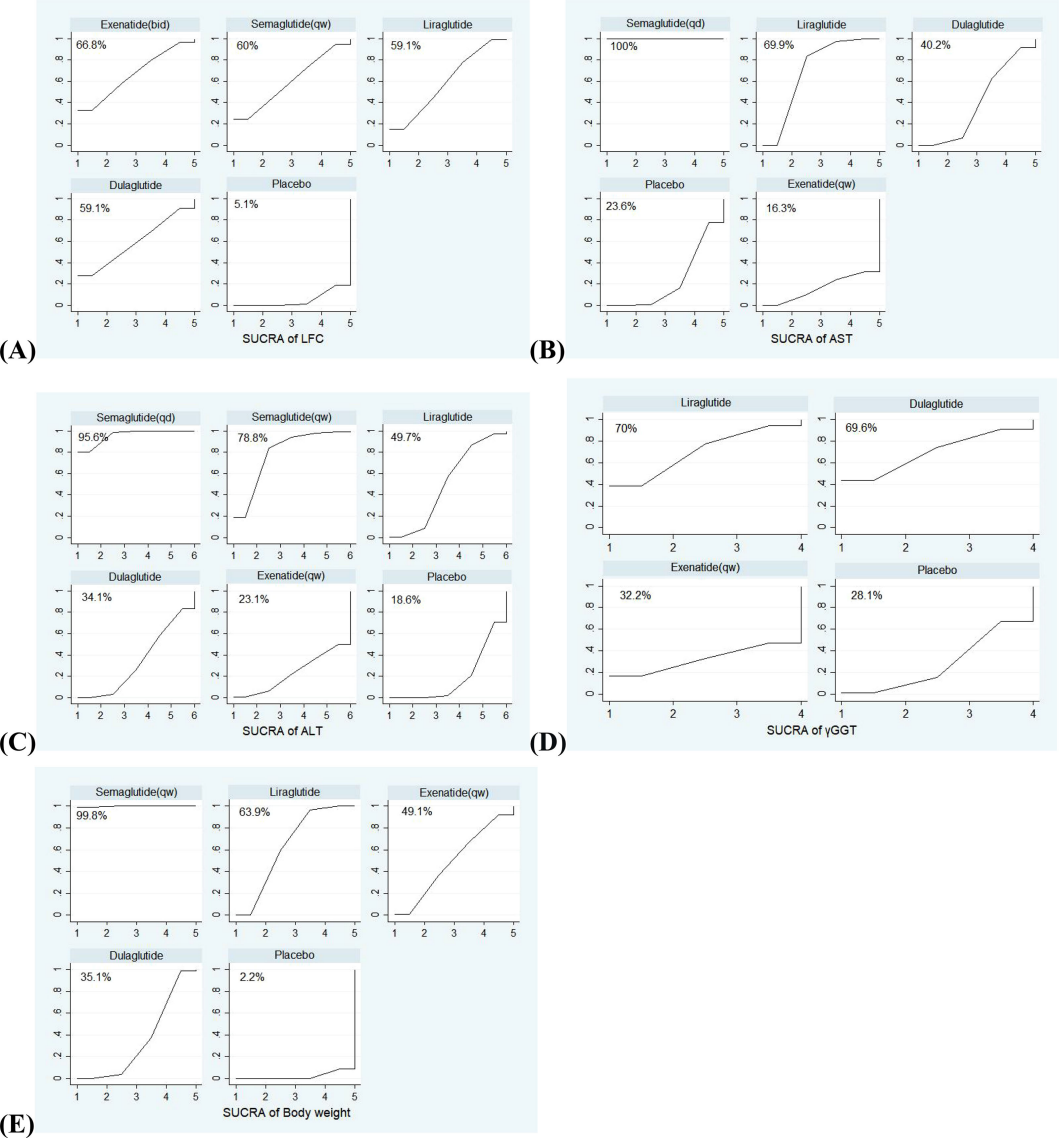
The SUCRA (surface under the cumulative ranking curve) of interventions for each outcome. The larger the surface under the curve, the more likely it is to be the best intervention. **(A)** SUCRA of LFC; **(B)** SUCRA of AST; **(C)** SUCRA of ALT; **(D)** SUCRA of γGGT; **(E)** SUCRA of Body weight.

**Figure 3:**
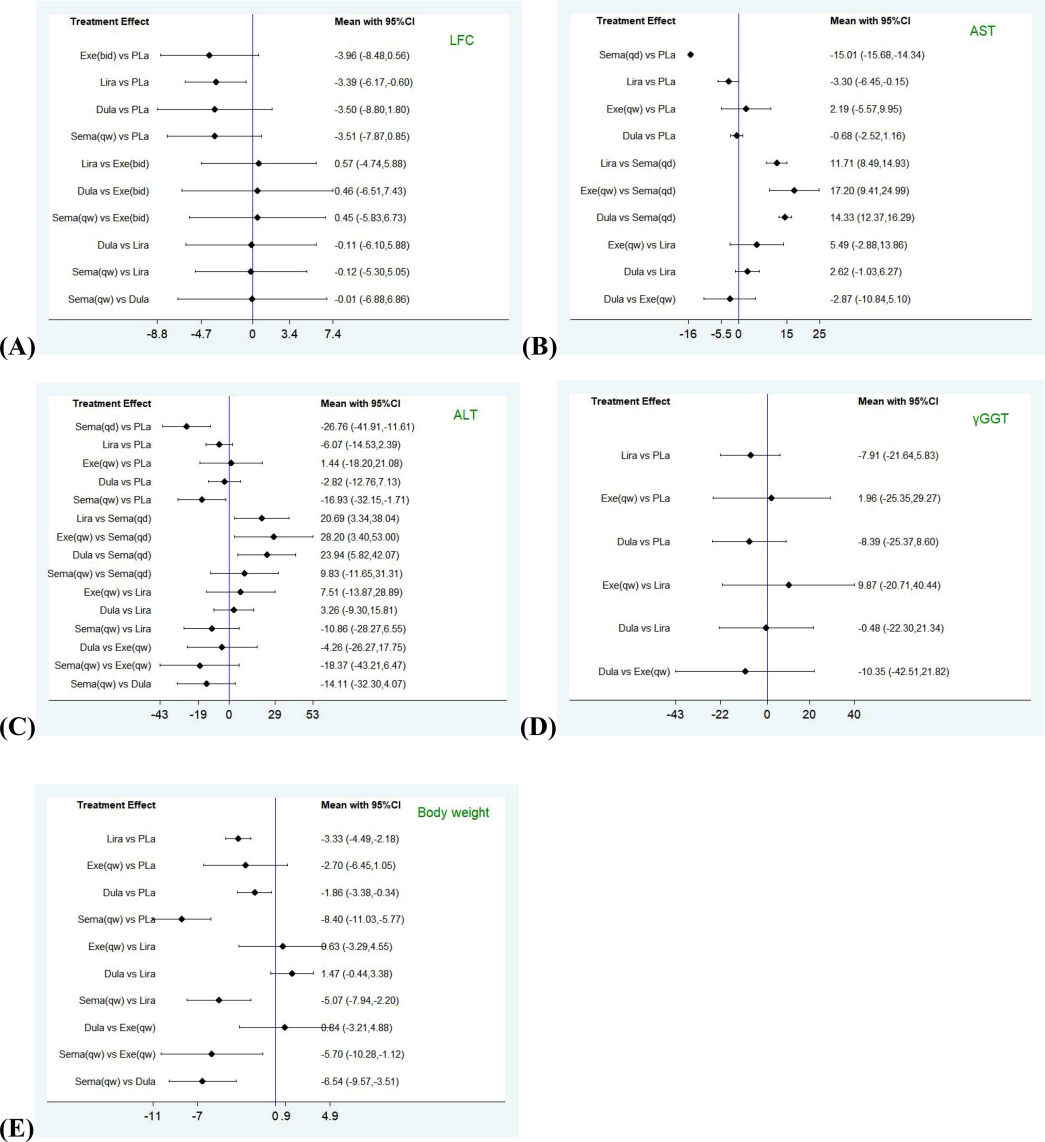
Forest plots comparing pairwise interventions for each outcome (LFC, AST, ALT, γGGT, Body weight). LFC, liver fat content; AST, aspartate aminotransferase; ALT, alanine aminotransferase; γGGT, γ-glutamyl transferase. **(A)** Forest plot comparing pairwise interventions for LFC; **(B)** Forest plot comparing pairwise interventions for AST; **(C)** Forest plot comparing pairwise interventions for ALT; **(D)** Forest plot comparing pairwise interventions for γGGT; **(E)** Forest plot comparing pairwise interventions for Body weight.

First, we constructed network plots of the outcomes to demonstrate all available evidence for each outcomes (**Figure 1**). Second, the outcomes we selected were all continuous variables, and therefore the mean and standard deviation (SD) changes from the baseline to the end and the sample size (n) were extracted for statistical analysis. The existing evidence only involved indirect comparison; therefore, the network graph had no closed loop and there was no need to examine the inconsistency of the outcomes. We employed SUCRA to evaluate the ranking of each intervention in each outcome (**Figure 2**). The higher the SUCRA value, the more likely the corresponding intervention to be regarded as the best treatment. “Zero” indicated that the treatment was the worst. The forest plots for each outcome were depicted in **Figure 3**, which shown the comparison between each intervention. The forest plots visually demonstrated the 95% confidence interval (CI) of the results of the pairwise comparison of interventions and whether they had any statistical significance. Finally, league plots were drawn based on SUCRA and the forest plots (**Figure 3**). The league plots ranked the effect of the intervention in each outcome from the best to the worst (**Table 3**). The results with statistical significance were highlighted in bold. The league plots more intuitively exhibited the effectiveness of each intervention. All of the abovementioned analyses were conducted by Stat17.0.

**Table 3:** League plots ranked the effect of the intervention in each outcome from best to worst.

(A) LFC						
Exenatide(bid)						
-0.45 (-6.73, 5.83)	Semaglutide(qw)					
-0.57 (-5.88, 4.74)	-0.12 (-5.3, 5.05)		Liraglutide			
-0.46 (-7.43, 6.51)	-0.01 (-6.88, 6.89)		0.11 (-5.88, 6.1)	Dulaglutide		
-3.96 (-8.48, 0.56)	-3.51 (-7.87, 0.85)		**-3.39** **(-6.17, -0.6)**	-3.5 (-8.8, 1.8)	Placebo	

(B) AST						
Semaglutide(qd)						
**-11.71** **(-14.93, -8.49)**	Liraglutide					
**-14.33** **(-16.29, -12.37)**	-2.62 (-1.03, 6.27)		Dulaglutide			
**-15.01** **(-15.68, -14.34)**	**-3.3** **(-6.45, -0.15)**		-0.68 (-2.52, 1.16)	Placebo		
**-17.20** **(-24.99, -9.41)**	-5.49 (-13.86, 2.88)		-2.87 (-10.84, 5.1)	-2.19 (-9.95, 5.57)	Exenatide(qw)	

(C) ALT						
Semaglutide(qd)						
-9.83 (-31.31, 11.65)	Semaglutide(qw)					
**-20.69** **(-38.04, -3.34)**	-10.86 (-28.27, 6.55)		Liraglutide			
**-23.94** **(-42.07, -5.82)**	-14.11 (-32.3, 4.07)		-3.26 (-15.81, 9.3)	Dulaglutide		
**-28.2** **(-53, -3.4)**	-18.37 (-43.21, 6.47)		-7.51 (-28.89, 13.87)	-4.26 (-26.27, 17.75)	Exenatide(qw)	
**-26.76** **(-41.91, -11.61)**	**-16.93** **(-32.15, -1.71)**		-6.07 (-14.53, 2.39)	-2.82 (-12.76, 7.13)	1.44 (-18.2, 21.08)	Placebo

(D) γGGT						
Liraglutide						
0.48(-21.34, 22.30)	Dulaglutide					
-9.87(-40.44, 20.71)	-10.35(-42.51, 21.82)		Exenatide(qw)			
-7.91(-21.64, 5.83)	-8.39(-25.37, 8.60)		1.96(-25.35, 29.27)	Placebo		

(E) Body weight						
Semaglutide(qw)						
**-5.07** **(-7.94, -2.2)**	Liraglutide					
**-5.7** **(-10.28, -1.12)**	-0.63 (-4.55, 3.29)		Exenatide(qw)			
**-6.54** **(-9.57, -3.51)**	-1.47 (-3.38, 0.44)		-0.84 (-4.88, 3.21)	Dulaglutide		
**-8.4** **(-11.03, -5.77)**	**-3.33** **(-4.49, -2.18)**		-2.7 (-6.45, 1.05)	**-1.86** **(-3.38, -0.34)**	Placebo	

Treatments are ranked according to their chances of being the best treatment. From left to right means it's less and less likely to be the best treatment. The leftmost intervention means the highest probability of being the best treatment, The rightmost intervention means the lowest probability of being the best treatment. The data in bold had statistical significance.

**Figure 4:**
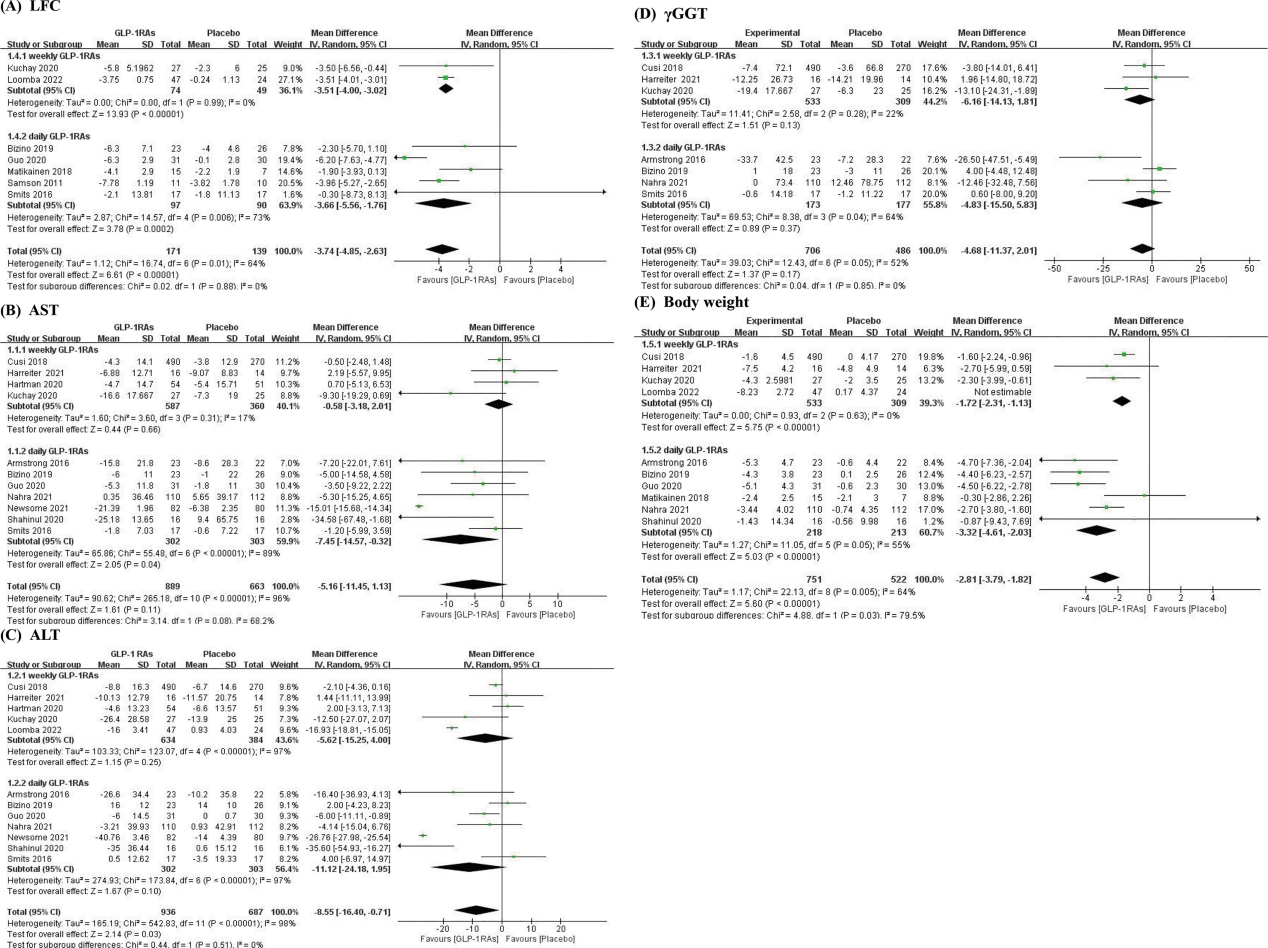
Forest plots of subgroup daily and weekly GLP-1RAs. **(A)** Subgroup forest plot of LFC; **(B)** Subgroup forest plot of AST; **(C)** Subgroup forest plot of ALT; **(D)** Subgroup forest plot of γGGT; **(E)** Subgroup forest plot of Body weight.

Then, we divided all studies with included outcomes into two subgroups of daily and weekly preparations, drew forest plots (**Figure 4**) using a random effects model to compared the mean difference (MD) between the two subgroups, and to observe which one was better in each outcome. The above analysis was performed by RevMan (version 5.4).

## 3 Results

### 3.1 Literature selection process and characteristics of studies

**Figure 5:**
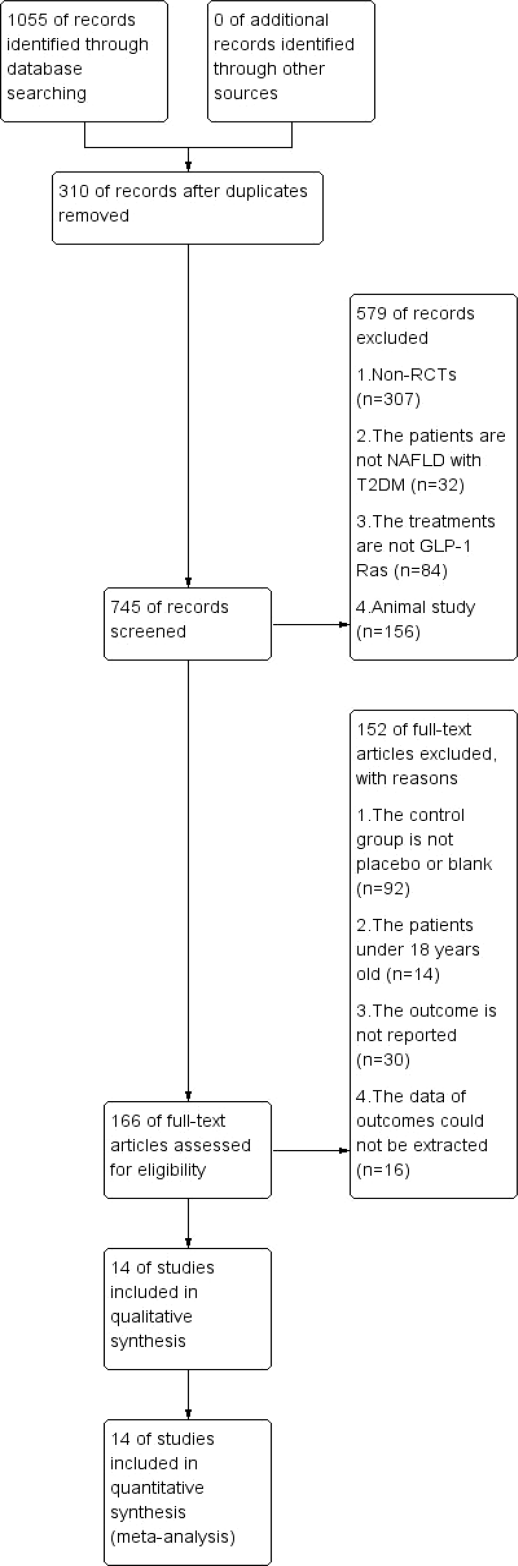
Literature selection process.

According to the search strategy, 1055 studies were searched from the following databases: PubMed, 224 studies; Embase, 649 studies; and Cochrane, 182 studies, and 310 duplicate references were removed. According to the inclusion and exclusion criteria, 14 RCTs were finally included in this NMA. The experimental group included five RCTs (12, 14–17) of weekly GLP-1RAs and nine RCTs (9, 18–25) of daily agents. The detailed literature selection process was shown in **Figure 5**.

As **Table 2** shown, the female to male ratio in the study population was approximately 1.19:1. Subjects from all over the world.

### 3.2 Quality assessment and evidence certainty assessment

**Figure 6:**
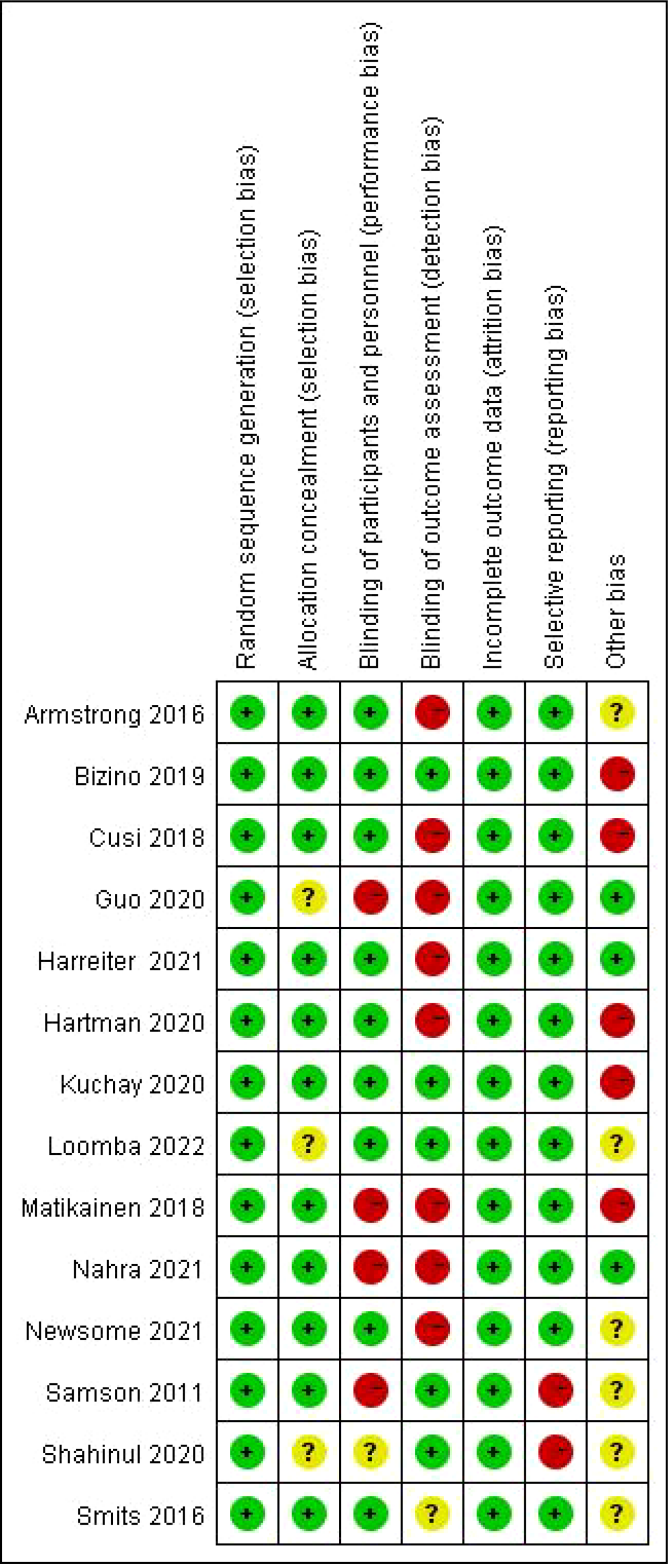
Quality assessment using the Cochrane risk assessment tool.

The quality of the included studies was assessed by the risk assessment of Cochrane review items. The following aspects were considered during the assessment: random sequence generation, allocation hiding, the blindness of participants and personnel, the blindness of result evaluation, incomplete result data, selective reporting, and other biases. The specific evaluation results were presented in **Figure 6**.

Using the GRADEprofiler to assess overall quality of evidence. The evaluation results were as follows: two outcomes were assessed as “low”, three outcomes were assessed as “moderate”. The assessment results were shown in **Table 4**.

**Table 4:** The quality of evidence assessment using the GRADE model.

outcome	Outcome important	Number of studies	Sample size	Evidence quality
LFC	critical	7	310	low
AST	critical	11	1552	moderate
ALT	critical	12	1623	moderate
γGGT	moderate	7	1194	low
body weight	important	10	1344	moderate

### 3.3 The outcomes

All experiments were included in this NMA, and the network evidence graphs of each outcome were shown in **Figure 1**. Among them, weekly GLP-1RA drugs in the treatment of patients with NAFLD mainly include semaglutide (qw), dulaglutide and exenatide (qw) and daily drugs include liraglutide, semaglutide (qd) and exenatide (bid). However, studies on other GLP-1RAs are scarce. The main outcomes we evaluated were LFC, ALT, and AST. Four drugs (except exenatide (bid) and semaglutide (qw)) showed the AST and five drugs (except exenatide (bid)) showed the ALT outcomes, and four drugs (except semaglutide (qd) and exenatide (qw)) showed the LFC outcome. The secondary outcomes were γGGT and body weight, whereas only three drugs (liraglutide, dulaglutide and exenatide (qw)) showed γGGT outcome, and all drugs, except exenatide (bid) and semglutide (qd), showed body weight outcome.

### 3.4 Network meta-analysis results

The SUCRA curves of interventions for outcomes were shown in **Figure 2**. Among five interventions (exenatide (bid), liraglutide, dulaglutide, semaglutide (qw) and placebo) evaluated for improving LFC, exenatide (bid) was the best (SUCRA = 66.8%, 59.1%, 59.1%, 60%, and 5.1%, respectively). Among five interventions (semaglutide (qd), liraglutide, dulaglutide, exenatide (qw), and placebo) evaluated for AST outcome, and six interventions (semaglutide (qd), liraglutide, dulaglutide, semaglutide (qw), exenatide (qw) and placebo) evaluated for ALT outcome, semaglutide (qd) was the most effective drug (SUCRA (AST) = 100%, SUCRA (ALT) = 95.6%). For AST, followed by liraglutide and dulaglutide (SUCRA (AST) = 69.9%, and 40.2%, respectively); For ALT, followed by semaglutide (qw) and liraglutide (SUCRA (ALT) = 78.8%, and 49.7%, respectively). Finally, the effects of four interventions (liraglutide, dulaglutide, exenatide (qw), and placebo) on γGGT were compared, and liraglutide was the most effective treatment (SUCRA (γGGT) = 70%), and the effects of five interventions (liraglutide, dulaglutide, semaglutide (qw), exenatide (qw), and placebo) on body weight were compared, semaglutide (qw) seemed better than liraglutide (SUCRA (body weight) = 99.8% *vs* 63.9%).

### 3.5 Subgroups results

The forest plots were shown that in all outcomes except γGGT, the daily preparations seemed more effective than weekly ones. The result of LFC in daily GLP-1RAs group was MD = -3.66, 95% CI [-5.56, -1.76] and in weekly GLP-1RAs group, it was MD = -3.51, 95% CI [-4, -3.02], p=0.88. As to AST and ALT, the results in daily GLP-1RAs group versus weekly GLP-1RAs group were AST: MD = -7.45, 95% CI [-14.57, -0.32] versus MD = -0.58, 95% CI [-3.18, 2.01], p=0.08 and ALT: MD = -11.12, 95% CI [-24.18, 1.95] versus MD = -5.62, 95% CI [-15.25, 4], p=0.51. The result of Daily GLP-1RAs group also was better than weekly one in body weight (MD = -3.32, 95% CI [-4.61, -2.03] *vs* MD = -1.72, 95% CI [-2.31, -1.13], p=0.03). However, the result of γGGT showed contrary to other outcomes (MD _daily_ = -4.83, 95% CI [-15.5, 5.83] *vs* MD _weekly_ = -6.16, 95% CI [-14.13, 1.81], p=0.85).

## 4 Discussion

In this NMA, we evaluated GLP-1RAs in the treatment of NAFLD to explore the effectiveness of the long-term use of weekly and daily preparations in improving LFC and liver enzymes involved in NAFLD. In the NMA, the subgroup results and SUCRA showed that the daily agents ranked ahead of the weekly agents with respect to primary outcomes. Though SUCRA showed that semaglutide (qw) was better than other agents on body weight, the subgroup results showed that daily group might be the most effective as a whole. Therefore, we speculate that daily agents show greater promise in NAFLD and T2DM treatment. Furthermore, the daily semaglutide seemed to improve ALT more than the weekly semaglutide, which further validated the conclusion.

Presently, NAFLD is often considered a metabolic disorder associated with liver diseases, and liver steatosis is probably closely related to insulin resistance and T2DM (28). Increased fat content and insulin resistance can lead to liver inflammation and fibrosis (29). A meta-analysis of six RCTs shows that liraglutide can improved liver steatosis (8). Moreover, liraglutide can improve liver metabolic dysfunction and insulin resistance which play a role in NASH pathogenesis (30). Therefore, we can potentially use GLP-1RAs to treat NAFLD with T2DM.

Although liver biopsy is the gold standard for NAFLD diagnosis, it is not widely used because of its invasiveness. Therefore, researchers have proposed non-invasive examinations instead to diagnose NAFLD and evaluate therapeutic effects. For example, many meta-analyses use LFC to evaluate the improvement of patients with NAFLD (31, 32). Serum biomarkers, such as ALT and AST, are the most common non-invasive tests to assess liver diseases and are commonly used as the clinical indicators of hepatocyte injuries (33). A 6-month, double-blind, and placebo-controlled study shows that lower ALT levels were associated with LFC (34). Therefore, our primary outcomes for assessing GLP-1RA efficacy were LFC and ALT and AST levels. Furthermore, a systematic review included 23 RCTs of the effects of lifestyle interventions on liver steatosis and shows that reduce LFC and lowered liver transaminase levels are strongly associated with weight loss (35). A 5%–10% weight loss resulted in a 40%–80% reduction in liver fats in patients without diabetes and with type 2 diabetes (36). Thus, we used body weight as a secondary outcome in the present NMA.

The subgroup results showed that the daily preparations might be superior to the weekly preparations with respect to primary outcome. And SUCRA showed that semaglutide (qd) might be the best GLP-1RAs among six GLP-1RAs included in our NM. The efficacy of semaglutide (qd) was markedly superior in terms of ALT and AST. A 2019 study shows that semaglutide significantly reduces ALT levels (37), and an RCT by Anne Flint et al. published in 2021 shows that semaglutide significantly improves ALT and AST levels (38). Second, the daily GLP-1RAs significantly reduced LFC and body weight compared with the weekly agents. A 24-week RCTs show that exenatide (bid) can reduce the primary outcome, LFC (10). Although semaglutide (qw) also reduced LFC, the SUCRA values showed that it was slightly less likely to be the optimal treatment than exenatide (bid). However, a NMA compared efficacy and safety of 8 GLP-1RAs show that exenatide (bid) have an increased risk of adverse events withdrawals compared to semaglutide (qw) (39). For body weight, a study including 387 participants found that weight loss with semaglutide (qw) was significantly greater than that with liraglutide (40). And two meta-analyses showed that more significant weight loss was observed after liraglutide intervention than dulaglutide and other GLP-1RA interventions (41, 42). Semaglutide and liraglutide induce weight loss by lowering energy intake (43, 44), but semaglutide can also reduce weight by reducing appetite (44), which is not obvious in liraglutide (43), this may be the reason why semaglutide is more significant in weight loss. To summarize, daily preparations may be better in the treatment of NAFLD with T2DM. Of course, due to the small number of weekly agents studies included, more weekly agents versus placebo RCTs are needed to validate our results.

### 4.1 Strengths and limitations

GLP-1RAs have been a popular hypoglycemic drug in recent years. Apart from hypoglycemic and weight loss effects, GLP-1RAs are also of great research value in NAFLD. However, no studies have compared the efficacy of daily and weekly GLP-1RA treatments for NAFLD with T2DM yet. Therefore, we adopted the NMA method to comprehensively analyze the effect of several commonly used GLP-1RAs on the reduction of LFC, liver enzymes, and body weight in patients with NAFLD and T2DM and to obtain an optimal treatment. However, we included only five studies on the weekly agents, which was limited in number and may lead to weak evidence, thus RCTs including more studies on weekly agents *vs*. placebo are needed to validate the present results. Moreover, due to the lack of direct comparative studies of the two GLP-1RAs, we cannot analyze inconsistent. The league plots showed a comparison between liraglutide and the placebo, showing that the major outcome, LFC, was statistically significant; however, the rest of the results were not statistically significant, which might be because of the small sample size. And there is only one study of semaglutide(qw), thus more studies of weekly semaglutide are needed to compare with daily exenatide.to assess which is superior in LFC. In the future, more large-sample, head-to-head RCTs are required to confirm these findings.

## 5 Conclusion

We integrated the evidence on GLP-1RAs for NAFLD with T2DM treatment and concluded that the daily preparations were superior to the weekly preparations with respect to primary outcome. We found that the daily GLP-1RAs semaglutide among the six GLP-1RAs ((exenatide (bid), liraglutide, semaglutide (qd), dulaglutide, semaglutide (qw), exenatide (qw)) might be the most effective treatment options for NAFLD. This conclusion may provide a basis for clinicians to treat NAFLD with T2DM.

## Data availability statement

The original contributions presented in the study are included in the article/supplementary material. Further inquiries can be directed to the corresponding author.

## Author contributions

XY and ZG contributed to the conception and design of the study. XY and CY searched the databases and screened the literature. XY and KD participated in data extraction. XY performed the statistical analysis. The first draft of this article was written by XY. All authors contributed to the article and approved the submitted version.
